# Lysozyme-Functionalized PVA/CMC/β-Cyclodextrin/Pectin Hydrogel Films for Strawberry Preservation and Flexible Sensing

**DOI:** 10.3390/molecules31193377

**Published:** 2026-09-23

**Authors:** Yan Pan, Mingyao Qin, Yuan Xu, Yemei Chen, Xuejin Zhang, Bao Wang, Yao Liu, Lei Wang

**Affiliations:** 1 School of Grain Engineering and Nutritional Science, Jilin Business and Technology College, Changchun 130023, China; panweiyun1979@sina.com (Y.P.); 13783868292@163.com (M.Q.); 100000000@139.com (B.W.); lymuma1@163.com (Y.L.); 2 Key Laboratory of Molecular Enzymology and Engineering of Ministry of Education, School of Life Sciences, Jilin University, Changchun 130023, China; 19165402873@163.com (Y.X.); chenyemei2024@163.com (Y.C.); zxj010715163@163.com (X.Z.)

**Keywords:** PVA hydrogel film, lysozyme, strawberry preservation, flexible sensing, active food packaging

## Abstract

A multifunctional hydrogel film, denoted as PCDGPL, was developed for strawberry preservation, and its prospective flexible-sensing capability was evaluated. Distinct from previous systems centered on colorimetric freshness indication or chemically/photocrosslinked sensing networks, PCDGPL employs a physically crosslinked, freeze-thawed PVA/CMC/pectin/β-CD matrix to couple lysozyme-associated antibacterial preservation with NaCl-enabled tensile and compressive deformation responses; a full-formulation-minus-β-CD control further isolated the network-modulating contribution of β-CD. The hydrogel network was proposed to be stabilized by freeze–thaw-induced PVA physical junctions, together with multiple hydrogen-bonding interactions among CMC, pectin, and β-cyclodextrin. Glycerol improved flexibility and moisture retention, NaCl provided ionic conductivity, and lysozyme incorporation contributed to the antibacterial activity of the film. The optimized PCDGPL film showed balanced mechanical properties, stable cyclic tensile/compressive behavior, and reliable strain-dependent resistance responses under stretching and compression. It also exhibited effective antibacterial activity against *Escherichia coli* and *Staphylococcus aureus* with good cytocompatibility. In strawberry preservation tests, PCDGPL reduced weight loss to approximately 2.5% and decay rate to 15%, while maintaining approximately 97% of the initial firmness after 10 days. This work provides a promising hydrogel-based active packaging platform for strawberry preservation, together with a prospective capability for mechanical deformation sensing.

## 1. Introduction

Strawberry is one of the most popular fresh fruits owing to its attractive color, pleasant flavor, and high nutritional value [1]. However, strawberry fruit is highly perishable because of its soft texture, high moisture content, thin epidermal tissue, and intense postharvest metabolic activity. During storage, transportation, and retail display, strawberries are susceptible to mechanical damage, moisture loss, oxidative deterioration, and microbial contamination, which together lead to softening, decay, weight loss, and reduced commercial value [2,3]. Therefore, developing advanced packaging materials that can provide active preservation, moisture regulation, mechanical protection, and deformation monitoring is highly desirable for extending the shelf life of strawberries and improving the intelligence of fresh-fruit packaging.

Conventional petroleum-based plastic packaging films mainly serve as passive barriers to isolate fresh produce from the external environment and reduce water evaporation [4]. Although these materials can partially delay quality deterioration, their functions are generally limited, and they are unable to actively inhibit microbial growth or monitor mechanical disturbances during storage and transportation [5]. In addition, nondegradable plastic packaging raises environmental concerns and does not meet the increasing demand for sustainable food packaging [6,7]. In this context, active and intelligent packaging systems based on hydrophilic polymers and natural polysaccharides have attracted increasing attention because they can integrate preservation, protection, and sensing functions into a single packaging platform [8].

Hydrogel films are promising candidates for fresh-food packaging because of their three-dimensional hydrophilic networks, excellent flexibility, high water-retention capacity, and tunable physicochemical properties [9]. Compared with dry plastic films, hydrogel films can provide a softer and more conformable interface [10], which may help buffer mechanical stress and reduce local damage to fragile fruits such as strawberries. Meanwhile, their hydrated polymer networks can regulate the moisture microenvironment around packaged fruit and slow down surface dehydration [11]. Poly(vinyl alcohol) (PVA) is widely used for hydrogel film fabrication because of its good film-forming ability, biocompatibility, processability, and capacity to form physically crosslinked crystalline domains through repeated freeze–thaw treatment [12,13]. Nevertheless, pure PVA hydrogel films usually suffer from insufficient mechanical stability, limited functional activity, and inadequate performance under practical packaging conditions, which necessitates further structural reinforcement and functional modification [14].

Carboxymethyl cellulose (CMC) and pectin are natural polysaccharides with good hydrophilicity, biocompatibility, and potential for food packaging applications [15]. CMC contains abundant hydroxyl and carboxymethyl groups that can interact with PVA chains through multiple hydrogen bonds, thereby improving the structural stability and water-retention ability of hydrogel networks. Pectin, a plant-derived polysaccharide rich in hydroxyl and carboxyl groups, can further enhance the hydrogen-bonded network, improve toughness, and increase resistance to deformation [16]. The combination of freeze–thaw-induced PVA crystalline domains and CMC/pectin polysaccharide networks is expected to produce composite hydrogel films with balanced mechanical strength, flexibility, and moisture-regulating capability, which are essential for strawberry preservation packaging [12,15,16,17].

β-Cyclodextrin (β-CD), a cyclic oligosaccharide with a hydrophobic inner cavity and hydrophilic outer surface [18], can also participate in the construction of multifunctional hydrogel networks. The abundant hydroxyl groups on the outer surface of β-CD can form hydrogen-bonding interactions with PVA, CMC, and pectin, contributing to network stabilization and structural reinforcement [19]. In addition, the characteristic cavity structure of β-CD offers potential host-guest interactions with small molecules and may improve the functional adaptability of the hydrogel matrix [20]. Therefore, incorporating β-CD into a PVA/CMC/pectin hydrogel system can provide an additional molecular-level strategy for improving the integrity and application performance of the composite film.

Microbial contamination is one of the major factors responsible for postharvest decay of strawberries [21]. Lysozyme is a natural antimicrobial enzyme that can inhibit bacterial growth by damaging bacterial cell wall structures, and it has been widely considered a safe bioactive agent for food-related applications [22]. Incorporating lysozyme into hydrogel packaging films can endow the films with active antibacterial ability, thereby suppressing microbial proliferation on fruit surfaces and delaying spoilage [23]. Compared with direct application of free antimicrobial agents, immobilization or entrapment of lysozyme within a hydrogel matrix may facilitate more stable functional presentation while maintaining the structural integrity of the packaging film [24].

In addition to active preservation, flexible sensing is an important emerging function for intelligent food packaging. Strawberries are vulnerable to compression, vibration, stretching, and package deformation during handling, stacking, and transportation, and these mechanical disturbances may accelerate tissue damage and quality deterioration. Sodium chloride (NaCl), as a simple and food-compatible ionic component, can provide ionic conductivity within the hydrogel network, enabling resistance variation under external tensile or compressive deformation. Glycerol can act as both a plasticizer and humectant, improving film flexibility, moisture stability, and ion migration continuity [17]. The combination of NaCl and glycerol therefore provides an ionic and mechanical basis for evaluating the hydrogel film as a prospective flexible strain-sensing layer.

Several closely related PVA-based hydrogel systems have already combined food preservation, antibacterial activity, or sensing functions. Gong et al. developed a PVA/gallic acid/lysozyme hydrogel film for blueberry preservation, although electrical deformation sensing was not included [25]. Yu et al. prepared an anthocyanin-containing SHNC/PVA/chitosan hydrogel film that combined salmon preservation with pH/NH_3_-responsive colorimetric freshness indication [26]. Chen et al. reported a photocrosslinked PVA-p(AA-AM)-PA-Fe^3+^ hydrogel for strawberry preservation and multimodal mechanical sensing [27]. Fan et al. developed a PVA/chitosan/cyclodextrin supramolecular double-network hydrogel with antibacterial activity and high strain sensitivity, but without validation in a food-preservation system [28]. Against these precedents, PCDGPL is not positioned as having the highest absolute mechanical strength or gauge factor. Its specific distinction lies in a physically assembled, freeze–thawed PVA/CMC/pectin/β-CD network in which lysozyme provides antibacterial activity, the hydrated polysaccharide/glycerol matrix regulates moisture, and NaCl provides ionic conduction for tensile and compressive deformation responses. The specific contribution of β-CD was further evaluated using a full-formulation-minus-β-CD control. A quantitative comparison with closely related systems is provided in Tables S1 and S2. In this study, a lysozyme-functionalized PVA/CMC/β-CD/NaCl/glycerol/pectin composite hydrogel film, denoted as PCDGPL, was fabricated through a simple freeze-thaw strategy for active strawberry preservation and flexible strain sensing. The hydrogel film was designed on the basis of a freeze–thawed PVA framework with proposed physical junctions, together with CMC and pectin as hydrogen-bonded polysaccharide reinforcing components, β-CD as a network-stabilizing and function-adaptable component, lysozyme as an antimicrobial agent, and NaCl/glycerol as ionic conductive and plasticizing components. The structure, mechanical properties, antibacterial activity, biocompatibility, water-retention ability, electrical/strain-sensing performance, and strawberry preservation effect of the composite hydrogel films were systematically investigated. This work provides a feasible strategy for developing multifunctional hydrogel-based packaging materials that integrate active antibacterial preservation, moisture regulation, and flexible protection, while also exhibiting prospective capability for mechanical-deformation sensing. The preparation process and potential applications of the PCDGPL hydrogel film are schematically illustrated in Figure 1.

## 2. Results

### 2.1. Characterization of Composite Hydrogels

To characterize the composition-dependent structural features of the PCDGPL hydrogels, cross-sectional morphology and intermolecular interactions were examined by SEM and FTIR. As shown in Figure 2a, all freeze-dried hydrogels exhibited porous cross-sectional structures, whereas the pore morphology varied with pectin content. PCDGPL-Pec_0_ and PCDGPL-Pec_0.1_ showed relatively compact and heterogeneous networks. In contrast, PCDGPL-Pec_0.2_ exhibited a more uniform and interconnected porous structure. Further increasing the pectin content resulted in enlarged and irregular pores, and locally collapsed regions became more evident. At 0.4 g pectin, dense and aggregated structures were observed.

The FTIR spectra of the hydrogels are shown in Figure 2b. All samples displayed a broad absorption band at 3200–3500 cm^−1^, corresponding to O–H stretching vibrations. With increasing pectin content from 0 to 0.4 g, the profile and position of this band varied, and the O–H band of PCDGPL-Pec_0.4_ showed a slight shift toward lower wavenumbers. In comparison, the C–H stretching band at 2900–3000 cm^−1^ exhibited relatively limited variation. The bands at 1590–1650 and 1400–1450 cm^−1^, corresponding to asymmetric and symmetric COO^−^ stretching vibrations, respectively, also showed composition-dependent variations. Changes were additionally observed in the 1000–1200 cm^−1^ region associated with C–O and C–O–C stretching vibrations. No distinct new absorption bands were detected after pectin incorporation.

### 2.2. Mechanical Properties of Composite Hydrogel Films

The tensile properties of different hydrogel formulations are presented in Figure 3a. Among the control samples, the PVA-Pec-NaCl-Gly hydrogel exhibited the highest tensile performance, with a tensile strength of approximately 0.9 MPa and an elongation at break of approximately 530–550%. The PVA-CMC-NaCl-Gly hydrogel showed substantially lower tensile strength, whereas the PVA-CD-NaCl-Gly hydrogel exhibited intermediate tensile performance. The preliminary multicomponent PVA-CMC-CD-Pec-Lys-NaCl-Gly formulation showed a tensile strength of approximately 0.34–0.35 MPa and an elongation at break of approximately 370–390%.

The effect of pectin content on tensile performance is shown in Figure 3b. Both tensile strength and elongation at break initially increased and subsequently decreased with increasing pectin content. The hydrogel containing 0.2 g pectin exhibited the highest overall tensile performance, with a tensile strength of approximately 0.36 MPa and an elongation at break of approximately 460–480%. At 0.4 g pectin, both tensile strength and elongation at break were significantly lower than those of the 0.2 g pectin group (*p* < 0.05).

As shown in Figure 3c, the Young’s modulus of the pectin-free hydrogel was approximately 0.036 MPa and increased to approximately 0.056 MPa after the addition of 0.1 g pectin. The Young’s modulus values of the 0.2, 0.3, and 0.4 g pectin groups were approximately 0.036, 0.035, and 0.030 MPa, respectively. Thus, the 0.1 g pectin group exhibited the highest initial stiffness, whereas the 0.2 g pectin group showed the highest tensile strength and toughness. Statistical analysis confirmed significant effects of pectin content on Young’s modulus and toughness (*p* < 0.05).

To evaluate the specific contribution of β-CD, the complete PCDGPL formulation was compared with the corresponding full-formulation-minus-β-CD control (PCDGPL-CD). As shown in Figure S1, the addition of β-CD significantly increased tensile strength from approximately 0.16 to 0.36 MPa (*p* < 0.05), whereas fracture strain remained at approximately 480–490% without a significant difference (*p* > 0.05). The compressive stress at 80% strain increased from approximately 0.30 to 2.4 MPa after β-CD incorporation (*p* < 0.05).

The cyclic tensile behavior of the optimized PCDGPL hydrogel is shown in Figure 3d–f. With increasing maximum strain from 50% to 250%, tensile stress progressively increased and reached approximately 0.18 MPa at 250% strain. During 10 consecutive loading–unloading cycles, the peak tensile stress remained within approximately 0.34–0.38 MPa, with an average value of approximately 0.36 MPa. Hysteresis energy decreased after the initial cycle and subsequently remained relatively stable.

The compressive properties are presented in Figure 3g–i. All samples exhibited nonlinear stress–strain behavior with a marked increase in compressive stress at high strain. Among the different pectin contents, the 0.3 g pectin group exhibited the highest compressive stress at 80% strain, reaching approximately 4.5 MPa. During 10 consecutive compression cycles, the optimized PCDGPL hydrogel exhibited peak compressive stresses of approximately 0.554–0.639 MPa. After the initial cycle, the hysteresis energy remained at approximately 0.006 MJ m^−3^.

### 2.3. Electrical Sensing Performance of the Optimized PCDGPL Hydrogel Film

The ionic conductivity of the optimized PCDGPL hydrogel was measured to be 1.072 S m^−1^. As shown in Figure 4a, the relative resistance change (Δ*R*/*R*_0_) increased continuously with tensile strain over the range of 0–300%. The strain-response curve exhibited two approximately linear regions, with gauge factors (GF) of 0.367 and 0.671, respectively.

The stepwise tensile response is presented in Figure 4b. When tensile strain was sequentially increased from 0% to 50%, 100%, 150%, 200%, and 250%, the Δ*R*/*R*_0_ signal increased stepwise and subsequently decreased during unloading. Distinguishable response levels were observed for the different applied strains. The response and recovery times were approximately 400 and 420 ms, respectively (Figure 4c).

Cyclic sensing performance was further evaluated under different stretching rates and strain levels (Figure 4d–f). Repeated tensile loading produced reproducible resistance responses, and the response amplitude increased with increasing applied strain. Under fixed-strain cycling and long-term repeated loading–unloading, the resistance signals retained relatively stable profiles.

The compression-responsive behavior is shown in Figure 4g–i. The relative resistance decreased with increasing compressive strain. Piecewise fitting of the compression-response curve yielded dimensionless compressive gauge factors (GFc) of approximately 2.82 and 0.492 in the low- and high-compression-strain regions, respectively. Repeated compression at 10%, 20%, and 30% strain generated distinguishable and repeatable signals. The response and recovery times under compression were approximately 400 and 300 ms, respectively.

### 2.4. Antibacterial Activity and Cytocompatibility of Composite Hydrogels

The antibacterial activity of the hydrogel films was evaluated against *E. coli* and *S. aureus*. As shown in Figure 5a, the blank control exhibited negligible antibacterial activity, whereas the hydrogel-film groups showed different degrees of growth inhibition. Among the lysozyme-free formulations, the PVA-CD-NaCl-Gly hydrogel exhibited a more evident inhibition zone than the other control formulations. The optimized PCDGPL hydrogel exhibited the largest total inhibition-zone diameters against both bacterial strains.

Quantitative analysis of the inhibition-zone diameters is shown in Figure 5c. For *E. coli*, PCDGPL exhibited significantly larger total inhibition-zone diameters than the PVA-CMC-NaCl-Gly and PVA-Pec-NaCl-Gly groups (*p* < 0.05), whereas no significant difference was observed between PCDGPL and PVA-CD-NaCl-Gly (*p* > 0.05). For *S. aureus*, PCDGPL showed a significantly larger inhibition-zone diameter than all lysozyme-free hydrogel formulations (*p* < 0.05).

The cytocompatibility of the hydrogel films was assessed using L929 fibroblasts. As shown in Figure 5b, most cells exhibited green fluorescence after treatment with hydrogel extracts, whereas relatively few dead cells with red fluorescence were observed. The quantitative cell-viability results in Figure 5d were consistent with the live/dead staining observations. The hydrogel-treated groups maintained relatively high cell viability after 24 h of culture, and the optimized PCDGPL hydrogel showed acceptable cytocompatibility under the tested conditions.

### 2.5. Packaging-Related Physical Properties and Strawberry Preservation

The packaging-related physical properties of the optimized PCDGPL hydrogel film were evaluated before the strawberry-preservation experiment. The film thickness ranged from approximately 150 to 300 μm (0.15–0.30 mm), and the equilibrium water content was approximately 88%. The swelling ratio increased rapidly during the initial immersion period and subsequently approached equilibrium, reaching approximately 815% after 7 days (Figure S2b). The measured water-vapor permeability was approximately 2.0 × 10−10 g m^−1^ s^−1^ Pa^−1^ (Figure S2a).

The strawberry-preservation performance of the different packaging films is shown in Figure 6. Representative photographs obtained on days 0, 1, 3, 5, 7, and 10 showed progressively increasing differences among the packaging treatments. At the later storage stages, strawberries packaged with PE exhibited pronounced surface darkening, shrinkage, and visible spoilage, whereas strawberries packaged with PCDGPL retained a relatively brighter red appearance and more intact surface morphology.

Quantitative analysis further demonstrated differences among the treatment groups. On day 10, the PE group exhibited a weight loss of approximately 12%, whereas the PCDGPL group showed the lowest weight loss of approximately 2.5%. The PCDGPL group also exhibited a significantly lower decay rate than the PE group and the other hydrogel-film groups (*p* < 0.05), with a final decay rate of approximately 15%. Firmness retention of the PCDGPL-packaged strawberries was approximately 97%, significantly higher than that of the other treatment groups on day 10 (*p* < 0.05). In addition, the PCDGPL group exhibited the lowest pH value, approximately 3.35, after storage (*p* < 0.05).

## 3. Discussion

### 3.1. Structure–Property Relationships in the PCDGPL Hydrogel Network

The SEM and FTIR results indicate that pectin content substantially influenced the organization of the multicomponent PCDGPL hydrogel network. The relatively uniform and interconnected porous morphology observed at 0.2 g pectin suggests that an appropriate pectin content favored a more homogeneous polymer network. In contrast, the enlarged and irregular pores observed at higher pectin contents may be related to the increased viscosity of the precursor solution and less uniform component distribution, which could interfere with the formation of a homogeneous freeze–thawed network.

The broad O–H absorption band and its composition-dependent shifts indicate changes in the hydrogen-bonding environment among the hydrogel components. Variations in the COO^−^ and polysaccharide fingerprint regions further support changes in intermolecular association involving pectin, PVA, CMC, and β-CD. The absence of distinct new absorption bands suggests that pectin incorporation occurred predominantly through physical intermolecular interactions rather than through the formation of new covalent functional groups [29,30].

Freeze–thaw-induced PVA crystallite formation is a well-established mechanism for physically crosslinked PVA hydrogels [12]. However, because no direct XRD or DSC characterization was performed in the present study, the contribution of PVA crystalline domains should be regarded as a proposed mechanism supported by previous studies rather than as directly confirmed structural evidence in the present multicomponent system.

### 3.2. Mechanical Reinforcement and Energy-Dissipation Mechanisms

The mechanical results demonstrate a concentration-dependent effect of pectin on the PCDGPL hydrogel network. Although the 0.1 g pectin formulation exhibited the highest Young’s modulus, the 0.2 g pectin formulation showed a better overall balance of tensile strength, elongation, and toughness. This difference indicates that maximum initial stiffness does not necessarily correspond to optimum overall mechanical performance. The abundant hydroxyl and carboxyl groups of pectin can participate in hydrogen bonding with PVA and other polysaccharides, thereby contributing to stress transfer and energy dissipation within the hydrogel network [15,16].

At excessive pectin contents, however, increased precursor viscosity and reduced component homogeneity may impair effective network organization. This interpretation is consistent with both the decline in tensile properties at 0.4 g pectin and the less homogeneous morphology observed by SEM. Interestingly, the 0.3 g pectin formulation exhibited the highest compressive stress, indicating that the formulation providing the best tensile behavior was not identical to that providing the highest compressive resistance. A moderately higher pectin content may therefore promote local network compactness under compression while simultaneously reducing tensile extensibility.

The full-formulation-minus-β-CD comparison further demonstrated a substantial contribution of β-CD to the load-bearing capability of the network. The marked increases in tensile strength and compressive stress after β-CD incorporation indicate that β-CD participates in the physical reinforcement of the composite network, most likely through its rigid cyclic structure and abundant hydroxyl groups. However, because direct host–guest binding experiments were not performed, the role of β-CD should be conservatively attributed to network modulation and intermolecular physical interactions rather than to a confirmed host–guest inclusion mechanism.

The hysteresis observed during tensile and compressive loading indicates the presence of energy-dissipation processes within the network [14,31]. These processes may involve reversible disruption and reconstruction of hydrogen bonds, rearrangement of polymer-chain entanglements, proposed freeze–thaw-induced PVA physical junctions, and glycerol-assisted dynamic interactions. The reduction in hysteresis energy after the first cycle followed by relatively stable subsequent cycles suggests that part of the weak physical network was rearranged during initial deformation and subsequently reached a more reproducible dynamic state.

### 3.3. Ionic Sensing Mechanism and Performance Characteristics

The sensing behavior of PCDGPL primarily originates from deformation-induced changes in ionic transport pathways within the hydrated NaCl-containing hydrogel network [32]. NaCl provides mobile ions, whereas the hydrated polymer matrix serves as the ion-transport medium. During tensile deformation, elongation of the hydrogel increases the effective ion-transport distance and partially disrupts continuous conductive pathways, resulting in an increase in resistance. At higher tensile strains, the greater structural deformation is consistent with the increase in GF from 0.367 to 0.671.

The GF values obtained in the present study were lower than the GF of 1.70 reported for a CNF/PVA/ZnSO_4_ ionic hydrogel over 0–200% strain [33] and the GF range of 1.31–9.21 reported for a strain-concentrated PVA-based ionic hydrogel over 0–100% strain [34]. Therefore, PCDGPL should not be positioned as an exceptionally high-sensitivity strain sensor. Its main sensing features are the relatively wide detectable tensile-strain range, distinguishable deformation-dependent resistance responses, rapid response/recovery behavior, and reproducibility during cyclic loading.

Under compression, the opposite resistance trend was observed. Compression reduces the distance between conductive pathways and increases contact within the hydrated network, resulting in a decrease in resistance. The higher GFc observed in the low-compression-strain region may reflect a greater relative change in ion-transport pathways during the initial stages of network densification. At higher compression, further structural compaction produces a comparatively smaller change in resistance per unit strain.

Although the hydrogel demonstrated reliable tensile and compressive deformation sensing, the present experiments primarily establish its potential as a mechanical-deformation-sensing layer. Further integration with signal-acquisition components and direct validation under actual packaged-fruit transportation or handling conditions would be required before the sensing function could be considered a practical intelligent-packaging system [35,36].

### 3.4. Antibacterial Activity and Cytocompatibility

The antibacterial results indicate that the final PCDGPL formulation exhibited stronger bacterial-growth inhibition than most lysozyme-free control formulations. Lysozyme can inhibit bacterial proliferation through disruption of bacterial cell-wall structures [37,38], and its incorporation into the hydrogel therefore likely contributed substantially to the antibacterial activity of PCDGPL. Nevertheless, some lysozyme-free formulations also exhibited measurable baseline inhibition, indicating that the antibacterial performance of the final film cannot be attributed exclusively to lysozyme.

Differences between *E. coli* and *S. aureus* may be related to their distinct cell-envelope structures. The thick peptidoglycan layer of Gram-positive *S. aureus* is directly accessible to lysozyme-mediated degradation, whereas the outer membrane of Gram-negative *E. coli* may partially restrict lysozyme access to the underlying peptidoglycan layer [21,39]. This structural difference provides a plausible explanation for the stronger antibacterial response observed against *S. aureus*.

An important limitation is that the residual enzymatic activity of lysozyme after repeated freeze–thaw processing was not independently quantified. Therefore, the inhibition-zone results should be interpreted as evidence of the antibacterial activity of the final PCDGPL film rather than as direct confirmation that lysozyme retained a specific proportion of its original enzymatic activity.

The relatively high L929 cell viability and predominance of live cells in the fluorescence images indicate acceptable cytocompatibility of the hydrogel formulations. This result is consistent with the use of PVA, CMC, pectin, β-CD, glycerol, NaCl, and lysozyme and with the physical freeze–thaw crosslinking strategy, which avoids the introduction of highly toxic chemical crosslinkers.

### 3.5. Strawberry Preservation Mechanism, Performance Positioning, and Limitations

The improved strawberry-preservation performance of PCDGPL likely resulted from the combined effects of moisture regulation, physical protection, and antibacterial activity [40]. The high equilibrium water content and swelling capacity indicate that the hydrogel maintained a highly hydrated polymer network, whereas the measured WVP demonstrates that the film allowed controlled water-vapor transfer rather than functioning as a completely impermeable barrier. Together, these properties may help maintain a relatively humid microenvironment around the fruit and reduce excessive moisture loss during storage.

The lower weight loss and higher firmness retention of strawberries packaged with PCDGPL are consistent with improved moisture retention and delayed tissue deterioration [41,42,43,44]. Meanwhile, the reduced decay rate is consistent with the antibacterial activity of the final hydrogel film. Maintenance of a lower pH value may also reflect delayed spoilage-related metabolic changes during storage.

Nevertheless, because water migration between the hydrated film and the fruit was not independently quantified, the contribution of direct film-to-fruit moisture transfer cannot be completely excluded. The preservation results should therefore be interpreted as the overall moisture-regulation performance of the film–fruit packaging system rather than solely as suppression of physiological water loss from the fruit.

Comparison with previously reported multifunctional PVA-based hydrogel systems further clarifies the positioning of PCDGPL. As summarized in Tables S1 and S2, PCDGPL does not necessarily exceed previously reported systems in absolute tensile strength or gauge factor. Its principal contribution instead lies in integrating lysozyme-associated antibacterial activity, moisture regulation, mechanical protection, and ionic tensile/compressive deformation responses within a relatively simple freeze–thawed PVA/CMC/pectin/β-CD network without an additional covalent crosslinker, photoinitiator, or metal-coordination step.

Because specimen geometries, storage conditions, and sensing protocols differ among published studies, the data summarized in Tables S1 and S2 should be regarded as benchmarking information rather than as a direct performance ranking. Likewise, the present sensing function should be conservatively described as a prospective mechanical-deformation-sensing capability rather than as a demonstrated freshness-monitoring function.

## 4. Materials and Methods

### 4.1. Materials

Poly(vinyl alcohol) (PVA, degree of hydrolysis: 87–89%, Mw: 31,000–50,000 g mol^−1^), sodium carboxymethyl cellulose (CMC), β-cyclodextrin (β-CD), pectin, sodium chloride (NaCl), glycerol, and lysozyme were purchased from Aladdin Reagent Co., Ltd. (Shanghai, China) and used without further purification. Deionized water was used throughout all experiments. Fresh strawberries with similar size, color, and maturity, and without visible mechanical damage or microbial spoilage, were purchased from a local market. Before the preservation experiment, strawberries were gently cleaned, air-dried at room temperature, and randomly divided into different packaging groups. *Escherichia coli* (*E. coli*) and *Staphylococcus aureus* (*S. aureus*) were used as representative Gram-negative and Gram-positive bacteria, respectively, to evaluate the antibacterial activity of the hydrogel films. Luria–Bertani broth and agar were used for bacterial culture and inhibition zone tests. L929 fibroblast cells were used to evaluate the cytocompatibility of the hydrogel films. Dulbecco’s modified Eagle’s medium, fetal bovine serum, phosphate-buffered saline, and live/dead staining reagents were used for cell culture and viability assays. All biological experiments were conducted under sterile conditions. All experiments were repeated at least three times to ensure reproducibility.

### 4.2. Preparation of Hydrogel Films

The hydrogel films were prepared by a simple freeze–thaw method [12]. Briefly, 6.0 g of PVA was dissolved in 20 mL of deionized water at 95 °C under continuous stirring for 1.5–2 h until a transparent and homogeneous solution was obtained. Separately, 0.7 g of CMC was dispersed in 30 mL of deionized water and stirred at 50 °C until completely dissolved. The PVA solution was then slowly added into the CMC solution under stirring to obtain a uniform PVA/CMC mixed solution. For the optimized hydrogel formulation, 0.5 g of β-CD, 0.8 g of NaCl, 0.6 g of glycerol, and 0.2 g of pectin were sequentially added into the PVA/CMC solution and stirred until fully dispersed. After the mixture was cooled to below 40 °C, 0.05 g of lysozyme was added and gently stirred to avoid thermal inactivation of the protein. The obtained precursor solution was degassed to remove air bubbles and then poured into Petri dishes. The samples were subjected to repeated freeze–thaw treatment, in which each cycle consisted of freezing at −20 °C for 12 h and thawing at room temperature for 12 h. After five freeze–thaw cycles, the optimized PCDGPL hydrogel film was obtained. Lysozyme was incorporated only after the precursor solution had cooled to below 40 °C to minimize thermal denaturation during mixing. However, the residual enzymatic activity of lysozyme after the five freeze-thaw cycles was not independently quantified in the present study.

For comparison, control hydrogel films with different compositions were prepared using the same procedure. All control formulations were adjusted to the same final volume as the optimized PCDGPL formulation. When CMC or another component was omitted, an appropriate amount of deionized water was added to maintain the same final volume, while the amounts of the remaining components and the freeze-thaw conditions were kept unchanged. The PVA-CMC-NaCl-Gly hydrogel was prepared by adding CMC, NaCl, and glycerol into the PVA matrix. The PVA-Pec-NaCl-Gly hydrogel was prepared by adding pectin, NaCl, and glycerol into the PVA matrix. The PVA-CD-NaCl-Gly hydrogel was prepared by adding β-CD, NaCl, and glycerol into the PVA matrix. In addition, pectin-gradient hydrogel films were prepared by varying the pectin content from 0 to 0.4 g while keeping the other components unchanged. A full-formulation-minus-β-CD control, designated PCDGPL-CD, was prepared by omitting 0.5 g of β-CD from the optimized PCDGPL formulation, while maintaining the same amounts of all other components, the same final volume by adding deionized water as needed, and identical freeze-thaw conditions. All prepared hydrogel films were stored in sealed containers before further characterization.

### 4.3. Characterization of Hydrogels

The structural characteristics of the hydrogels were analyzed by scanning electron microscopy (SEM) and Fourier-transform infrared spectroscopy (FTIR). For SEM observation, the hydrogel samples were subjected to solvent exchange, rapidly frozen in liquid nitrogen, and subsequently freeze-dried. The dried specimens were fractured to expose the cross-sections, sputter-coated with gold, and examined on an SEM5000X field-emission scanning electron microscope (CIQTEK Co., Ltd., Hefei, China). FTIR spectra were recorded on an IRTracer-100 Fourier transform infrared spectrometer (Shimadzu Corporation, Kyoto, Japan) to identify the characteristic functional groups and intermolecular interactions within the hydrogel networks.

### 4.4. Mechanical Tests

Mechanical properties were measured using a HY-0580 electronic universal testing machine (Shanghai Hengyi Testing Instruments Co., Ltd., Shanghai, China). The precursor solutions were poured into custom-made PTFE molds for general hydrogel preparation or into 1 mL syringe molds for preparing cylindrical tensile specimens. The tensile specimens had a diameter of approximately 3.5 mm, corresponding to an initial cross-sectional area of 9.6 mm^2^, and an initial gauge length of 10 mm. Tensile testing was performed at a stretching rate of 100 mm min^−1^. Cylindrical specimens with an initial diameter of 10 mm and a height of 5 mm were used for compression testing at a compression rate of 10 mm min^−1^. Tensile and compressive stresses were calculated using the initial cross-sectional areas of the corresponding specimens. Each test was independently repeated three times. Young’s modulus was determined from the slope of the initial linear region of the tensile stress–strain curve, whereas toughness was calculated by integrating the area under the tensile stress–strain curve.

### 4.5. Strawberry Preservation

Fresh strawberries with uniform size, color, and maturity and without visible mechanical damage or microbial spoilage were selected for the preservation experiment. The strawberries were gently rinsed with sterile deionized water and air-dried at room temperature. The samples were randomly divided into five groups: PE, PVA-CMC-NaCl-Gly, PCDGPL, PVA-CD-NaCl-Gly, and PVA-Pec-NaCl-Gly. For the preservation test, strawberries were packaged with different films and placed in sterile storage containers. The PE film was used as the conventional packaging control. All samples were stored at approximately 25 °C under ambient relative humidity (approximately 43% RH). The relative humidity was not actively controlled. Each treatment group consisted of 30 strawberries, divided into three independent replicates of 10 fruits each. At each predetermined imaging interval, three representative strawberries were randomly selected from each group for photography. The photographs were used only to illustrate representative changes in appearance and did not represent the complete experimental sample set. The three randomly selected strawberries were also evaluated individually for firmness using a texture analyzer (*n* = 3). Each strawberry was measured separately, and the mean of the three individual firmness values was used as the firmness value of the corresponding treatment group at that storage time. The appearance of strawberries was recorded using a digital camera at predetermined storage intervals, including day 0, day 1, day 3, day 5, day 7, and day 10. The preservation performance was evaluated by monitoring visual appearance, weight loss, decay rate, firmness retention, and pH changes during storage [3].

The weight loss rate was calculated separately for each independent replicate of 10 strawberries according to the following equation:

Weight loss (%) = [(*W*_0_ − *W_t_*)/*W*_0_] × 100 (1)

where *W*_0_ is the initial total mass of the strawberries within one independent replicate and *W_t_* is the corresponding total mass at the specified storage time.

The decay rate was determined by counting the strawberries showing visible mold growth, surface collapse, or severe soft rot within each independent replicate and was calculated as follows:

Decay rate (%) = *(N_d_*/*N_t_*) × 100 (2)

where *N_d_* is the number of decayed strawberries within one independent replicate and *N_t_* is the total number of strawberries in that replicate (*N_t_* = 10). For both weight loss and decay rate, the values obtained from the three independent replicates were used to calculate the mean ± SD (*n* = 3).

The firmness of individual strawberries was measured using a texture analyzer (TA.XTplus, Stable Micro Systems, Surrey, UK) equipped with a cylindrical flat-ended probe (5 mm in diameter). Each intact strawberry was placed horizontally and punctured at two opposite positions in the equatorial region without removing the peel. The pre-test, test, and post-test speeds were set at 1.0, 1.0, and 5.0 mm s^−1^, respectively. The penetration depth was 5.0 mm, and the trigger force was 0.05 N. Firmness was defined as the maximum force recorded during penetration and was expressed in newtons (N). The mean value obtained from the two measurement positions was used as the firmness of each fruit. At each sampling time, three strawberries, one randomly selected from each independent replicate, were measured.

Firmness retention (%) = (*F_t_*/*F*_0_) × 100 (3)

where *F*_0_ and *F_t_* represent the mean firmness values obtained from the three individually measured strawberries at the initial and specified storage times, respectively.

For pH determination, strawberry pulp was analyzed according to a previously reported method with minor modifications [3]. Briefly, 10.0 g of pulp from each independently sampled strawberry was homogenized with 50 mL of deionized water (1:5, *w*/*v*). The homogenate was filtered, and the pH of the filtrate was measured at 25 °C using a calibrated digital pH meter (PHS-3C, INESA Scientific Instrument Co., Ltd., Shanghai, China). The pH meter was calibrated using standard buffer solutions of pH 4.00 and 7.00 before measurement. At each storage time, three strawberries, one randomly selected from each independent replicate, were analyzed separately.

### 4.6. Antibacterial Activity and Cytotoxicity Assays

The antibacterial activity of the hydrogel films was evaluated against *Escherichia coli* and *Staphylococcus aureus* using an agar-diffusion assay adapted from the standardized disk-diffusion procedure described by the Clinical and Laboratory Standards Institute (CLSI) [45]. All hydrogel film samples were cut into circular discs with a diameter of 1.0 cm and sterilized by ultraviolet irradiation before testing. The bacterial strains were cultured in Luria–Bertani broth at 37 °C until the logarithmic growth phase, and the bacterial suspensions were diluted to the concentration used for antibacterial evaluation. Subsequently, 100 μL of each bacterial suspension was uniformly spread onto sterile LB agar plates, after which the hydrogel discs were gently placed on the inoculated agar surface. An inoculated agar plate without a hydrogel disc was used as the blank control. After incubation at 37 °C for 24 h, the inhibition zones were photographed and measured. For the hydrogel-film groups, the reported inhibition-zone diameter represented the total diameter of the clear zone, including the 1.0 cm hydrogel disc. Accordingly, a measured diameter close to 1.0 cm indicated negligible inhibition beyond the disc edge. Because no disc was placed on the blank-control plate, the blank control was used only for qualitative comparison and was not included in the quantitative total-diameter analysis. Each experiment was independently performed in triplicate.

The cytotoxicity of the hydrogel films was evaluated using L929 fibroblast cells. Before cell experiments, the hydrogel films were sterilized by ultraviolet irradiation and then immersed in complete cell culture medium to obtain hydrogel extracts. L929 cells were cultured in Dulbecco’s modified Eagle’s medium supplemented with 10% fetal bovine serum and 1% penicillin-streptomycin at 37 °C in a humidified atmosphere containing 5% CO_2_. The cells were seeded in 96-well plates and incubated for 24 h to allow cell attachment. Then, the culture medium was replaced with the hydrogel extracts, and the cells were further incubated for 24 h. Cells cultured with fresh complete medium were used as the control group.

Cell viability was determined using a CCK-8 assay [46]. Briefly, after incubation with the hydrogel extracts, the culture medium was removed, and fresh medium containing CCK-8 reagent was added to each well. After incubation for an appropriate time, the absorbance at 450 nm was measured using a microplate reader. Cell viability was calculated according to the following equation:

Cell viability (%) = (*OD_sample_*/*OD_control_*) × 100 (4)

where *OD_sample_* and *OD_control_* represent the absorbance values of the hydrogel-treated group and the control group, respectively. In addition, live/dead staining was performed to visually evaluate cell viability [47]. After co-culture with the hydrogel extracts, the cells were stained with calcein-AM and propidium iodide according to the manufacturer’s instructions and observed using a fluorescence microscope. Live cells emitted green fluorescence, whereas dead cells emitted red fluorescence. All cytotoxicity experiments were conducted in triplicate.

### 4.7. Hydrogel Sensing Properties

The electromechanical sensing performance of the hydrogel films was evaluated by monitoring the relative resistance change under tensile and compressive deformation [48]. For tensile sensing measurements, cylindrical PCDGPL hydrogel specimens were prepared using 1 mL syringe molds. The specimens had an effective cross-sectional area of 9.6 mm^2^, corresponding to a diameter of approximately 3.5 mm, and an initial gauge length of 10 mm. For compression sensing measurements, cylindrical specimens with a diameter of 10 mm and a height of 5 mm were used. The initial dimensions of all specimens were measured before testing. Conductive wires were connected to both ends of each hydrogel strip using conductive tape to form a simple resistance-type sensor. The resistance signal was recorded using a digital multimeter, while mechanical deformation was applied using a universal testing machine. The relative resistance change was calculated according to the following equation:

Δ*R*/*R*_0_ = (*R* − *R*_0_)/*R*_0_ (5)

where *R*_0_ is the initial resistance of the hydrogel sensor and *R* is the resistance under applied deformation. For graphical presentation, Δ*R*/*R*_0_ was expressed as a percentage by multiplying the dimensionless value by 100%.

For tensile sensing tests, the hydrogel sensor was fixed between the clamps of the universal testing machine and stretched to different strains. The strain-dependent resistance response was recorded over a wide tensile strain range to evaluate the sensitivity of the hydrogel sensor [49]. The gauge factor (GF) was calculated using the following equation:

GF = (Δ*R*/*R*_0_)/*ε* (6)

where *ε* is the applied tensile strain. For compression sensing, the compressive gauge factor (GFc) was determined from the absolute slope of the linear fit of Δ*R*/*R*_0_ versus compressive strain (*ε_c_*). Because both Δ*R*/*R*_0_ and *ε_c_* are dimensionless quantities, GFc is also dimensionless. Stepwise tensile sensing tests were performed by gradually increasing the strain from 0% to 50%, 100%, 150%, 200%, and 250%, followed by stepwise release. The response and recovery times were determined by recording the resistance variation during rapid loading and unloading processes [33].

The cyclic tensile sensing stability was further investigated by repeatedly stretching and releasing the hydrogel sensor under fixed strain. To evaluate the effect of deformation rate, cyclic tensile tests were conducted at different stretching speeds. In addition, long-term sensing durability was assessed by recording the resistance signals during 100 continuous tensile loading-unloading cycles. Cyclic sensing tests under different tensile strains were also performed to examine the ability of the hydrogel sensor to distinguish different deformation levels [33,34]. For compression sensing tests, the hydrogel samples were placed between two compression plates, and the resistance change was recorded during compression and release. The compression-dependent resistance response was measured under different compressive strains. Stepwise or cyclic compression tests were conducted at selected compression strains to evaluate signal repeatability and strain discrimination capability. The response and recovery times under compression were also determined from the real-time resistance curves.

### 4.8. Packaging-Related Physical Properties of PCDGPL

The thickness of the optimized PCDGPL hydrogel film was measured at five randomly selected positions using a digital micrometer, and the thickness range was recorded.

For equilibrium water content determination, the equilibrium hydrated mass *W_e_* of the PCDGPL film was recorded. The sample was subsequently dried to a constant mass *W_d_*, and the EWC was calculated according to:

EWC (%) = (*W_e_* − *W_d_*)/*W_e_* × 100 (7)

For swelling measurement, the dried PCDGPL film with an initial mass of *W_d_* was immersed in deionized water at 25 °C. At predetermined intervals from 1 to 7 days, the sample was removed, and excess surface water was gently removed using filter paper before the swollen mass *W_s_* was recorded. The swelling ratio was calculated as follows:

SR (%) = (*W_s_* − *W_d_*)/*W_d_* × 100 (8)

The water-vapor permeability (WVP) of the hydrogel films was determined using a gravimetric desiccant-cup method. Defect-free film samples were sealed over the circular openings of permeation cups containing anhydrous calcium chloride, which maintained an approximately 0% relative humidity (RH) inside the cups. The exposed film diameter was 3.5 cm, corresponding to an effective permeation area (*A*) of 9.62 × 10^−4^ m^2^. The assembled cups were maintained at 25 °C under an ambient RH of approximately 43%, corresponding to the environmental conditions used in this study. The ambient RH was not actively controlled.

The cups were weighed at 24 h intervals using an analytical balance with a resolution of 0.0001 g. Measurements were continued until a linear increase in cup mass was obtained. The steady-state mass-gain rate (Δ*m*/Δ*t*) was determined from the slope of the linear portion of the mass-versus-time curve using linear regression, based on at least three consecutive measurement points. At 25 °C, the saturated water-vapor pressure was taken as 3.17 kPa. Because the RH values outside and inside the cups were approximately 43% and 0%, respectively, the water-vapor partial-pressure difference across the film was calculated as Δ*p* = 3.17 × (0.43 − 0) = 1.36 kPa. WVP was calculated according to the following equation:

WVP = Δ*m* × *d*/(*A* × Δ*t* × Δ*p*) (9)

where Δ*m*/Δ*t* is the steady-state mass-gain rate (g s^−1^), *d* is the mean film thickness (m), *A* is the effective permeation area (m^2^), and Δ*p* is the water-vapor partial-pressure difference across the film (Pa). The calculated WVP was expressed in g m^−1^ s^−1^ Pa^−1^.

The initial resistance (*R*_0_) of the undeformed PCDGPL hydrogel sensor was measured using a digital multimeter. Ionic conductivity was calculated according to:

*σ* = *L*/(*R*_0_
*A*) (10)

where *L* is the initial distance between the electrodes, *R*_0_ is the initial resistance, and *A* is the cross-sectional area of the hydrogel sample.

### 4.9. Statistical Analysis

Group-summary quantitative data are presented as the mean ± standard deviation (SD). Unless otherwise specified, quantitative experiments were independently performed in triplicate (*n* = 3), with each independent replicate treated as one experimental unit. Statistical significance was analyzed using one-way analysis of variance (ANOVA), followed by Tukey’s post hoc test. Differences were considered statistically significant at *p* < 0.05. Representative images and individual response curves were not treated as group-summary data.

## 5. Conclusions

In summary, a freeze-thawed PCDGPL hydrogel film was developed for strawberry preservation and flexible sensing. The hydrogel film combined mechanical stability, moisture retention, antibacterial activity, biocompatibility, and ionic conductivity through the synergistic effects of the freeze–thawed PVA framework with proposed physical junctions, polysaccharide hydrogen-bonding networks, glycerol, NaCl, and lysozyme. The optimized PCDGPL film exhibited stable tensile/compressive behavior, reliable strain-dependent resistance responses, and good cyclic sensing performance. It also showed effective antibacterial activity against *E. coli* and *S. aureus* with acceptable cytocompatibility. In strawberry preservation tests, PCDGPL reduced weight loss, delayed decay, maintained firmness, and stabilized pH compared with PE and other hydrogel formulations. The principal contribution of PCDGPL is therefore its compositionally resolved integration of lysozyme-associated antibacterial preservation, moisture regulation, and ionic deformation response within a physically crosslinked PVA/polysaccharide network, rather than superior absolute mechanical strength or sensing sensitivity. Therefore, this hydrogel film provides a promising active packaging platform for strawberry preservation and exhibits prospective deformation-sensing capability. Further integration and validation under actual fruit-packaging conditions will be required before its practical application as an intelligent packaging system.

## Figures and Tables

**Figure 1:**
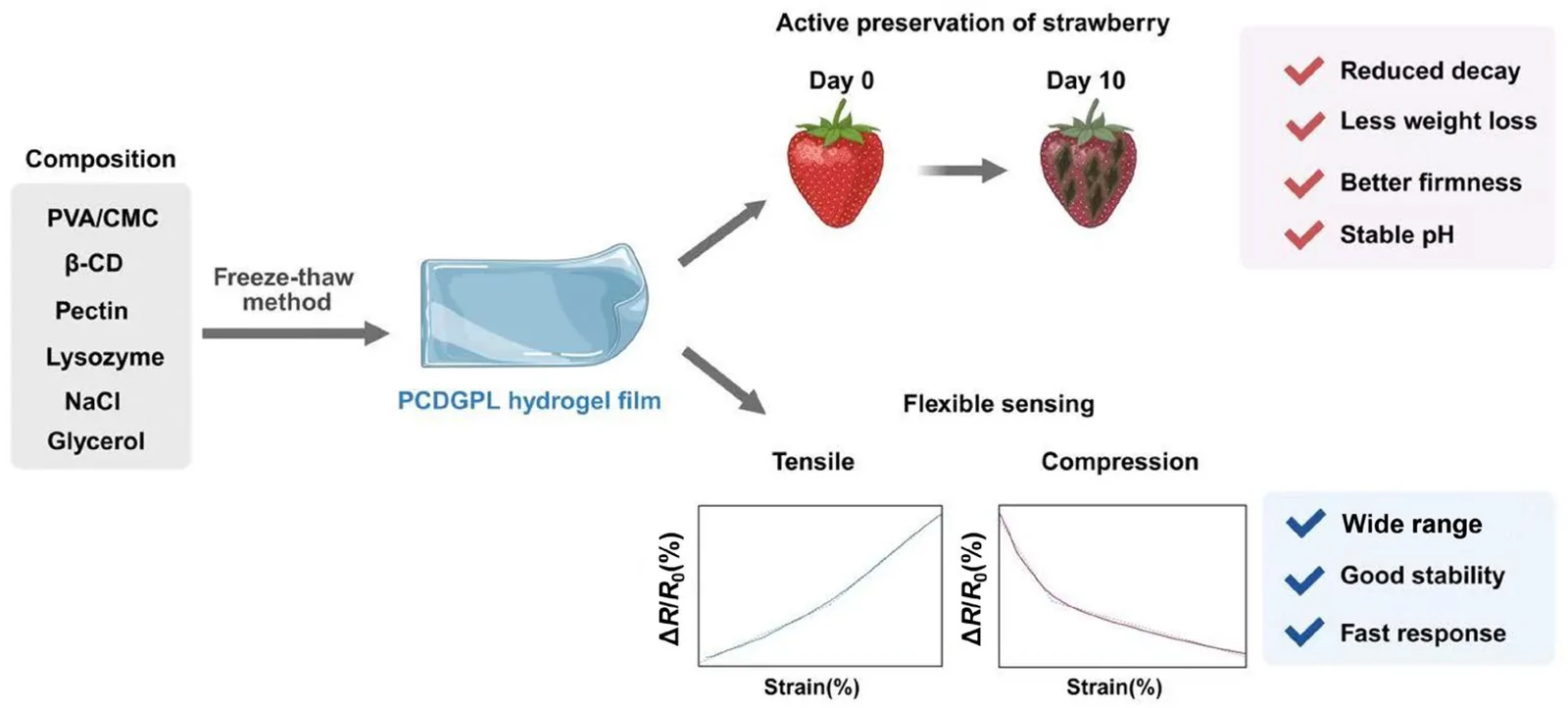
Schematic diagram of preparation of PCDGPL hydrogel composite film for strawberry preservation and flexible sensing.

**Figure 2:**
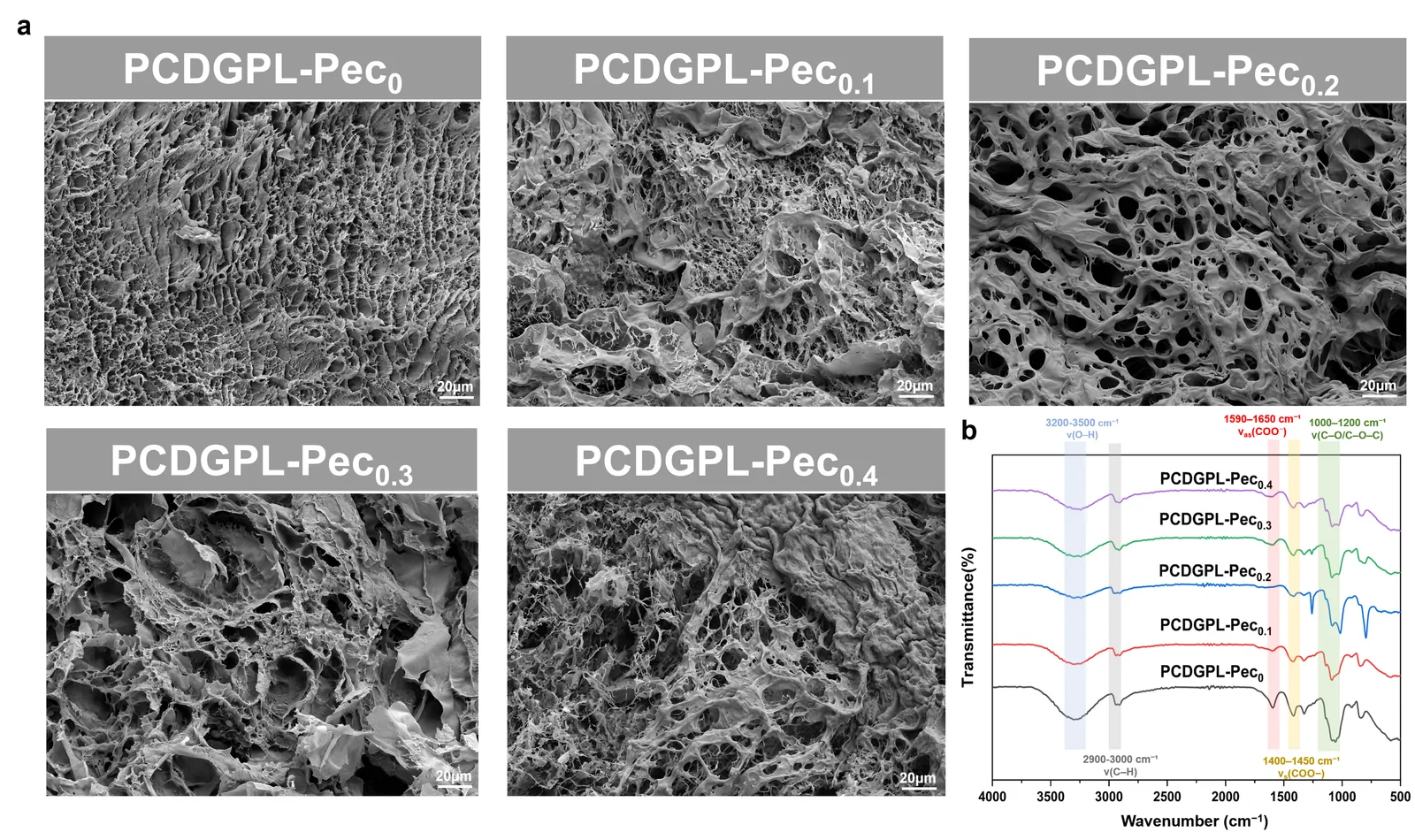
(**a**) SEM images showing the cross-sectional morphologies of PCDGPL-Pec hydrogels containing 0–0.4 g of pectin. (**b**) ATR-FTIR spectra of the corresponding PCDGPL-Pec hydrogels. The shaded regions indicate the principal absorption bands discussed in the text, including O–H stretching at 3200–3500 cm^−1^, C–H stretching at 2900–3000 cm^−1^, asymmetric and symmetric COO^−^ stretching at 1590–1650 and 1400–1450 cm^−1^, respectively, and C–O/C–O–C stretching at 1000–1200 cm^−1^. The spectra were vertically offset for clarity.

**Figure 3:**
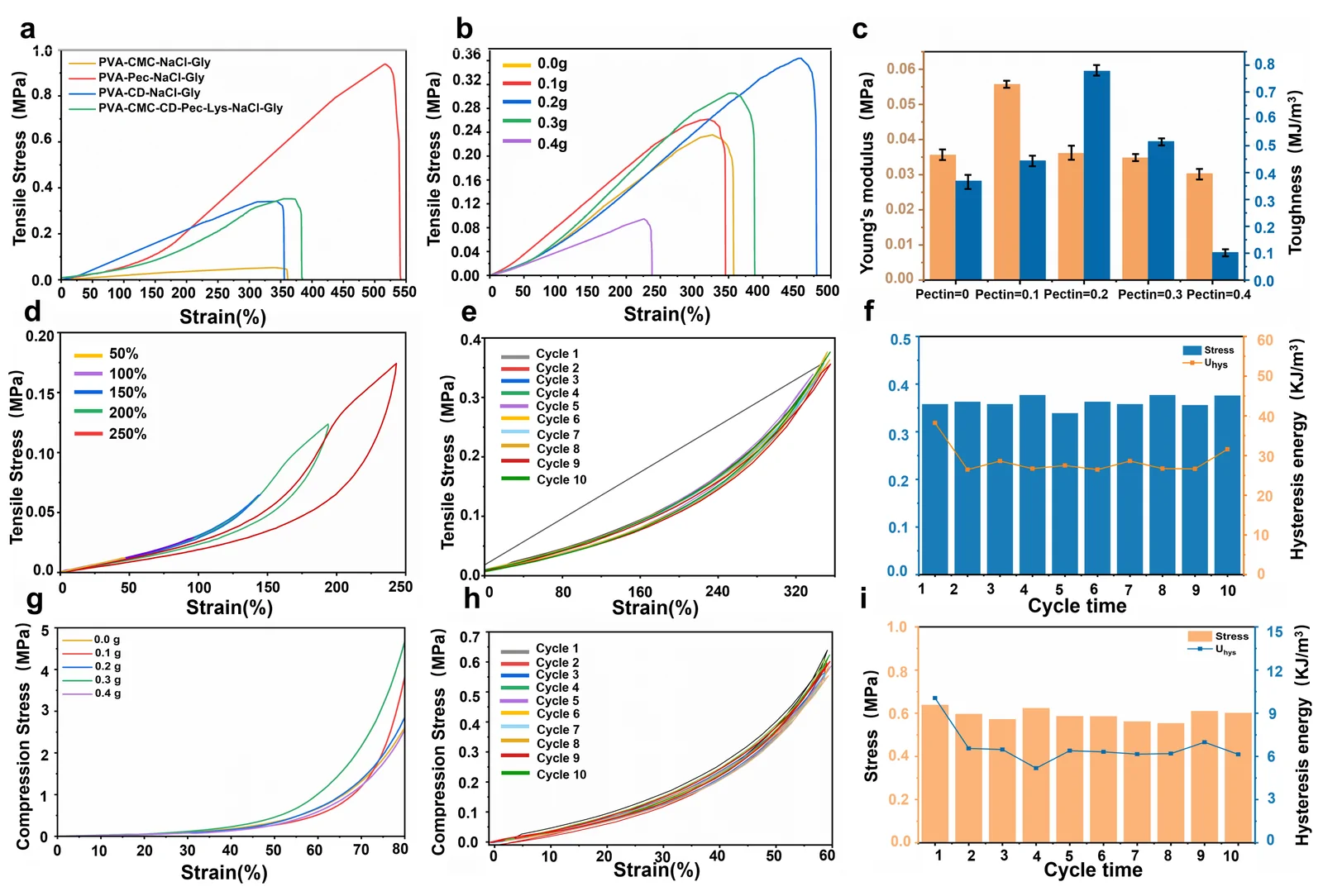
Mechanical properties of the hydrogels. (**a**) Tensile stress–strain curves of different control hydrogels and the preliminary multicomponent PVA-CMC-CD-Pec-Lys-NaCl-Gly formulation before pectin-content optimization. (**b**) Tensile stress–strain curves of hydrogels containing different pectin contents (0–0.4 g). (**c**) Young’s modulus (left *y*-axis) and toughness (right *y*-axis) of hydrogels containing different pectin contents. (**d**) Cyclic tensile stress–strain curves of the optimized PCDGPL hydrogel at different maximum strains. (**e**) Tensile stress–strain curves of the optimized PCDGPL hydrogel over 10 consecutive cycles. (**f**) Peak tensile stress and hysteresis energy of the optimized PCDGPL hydrogel during the 10 tensile cycles. (**g**) Compressive stress–strain curves of hydrogels containing different pectin contents. (**h**) Compressive stress–strain curves of the optimized PCDGPL hydrogel over 10 consecutive cycles. (**i**) Peak compressive stress and hysteresis energy of the optimized PCDGPL hydrogel during the 10 compression cycles. Data in panel (**c**) are presented as mean ± SD (*n* = 3). Statistical comparisons were performed using one-way ANOVA followed by Tukey’s post hoc test. The individual stress–strain and cyclic curves shown in the other panels are representative results from three independent measurements that exhibited comparable trends.

**Figure 4:**
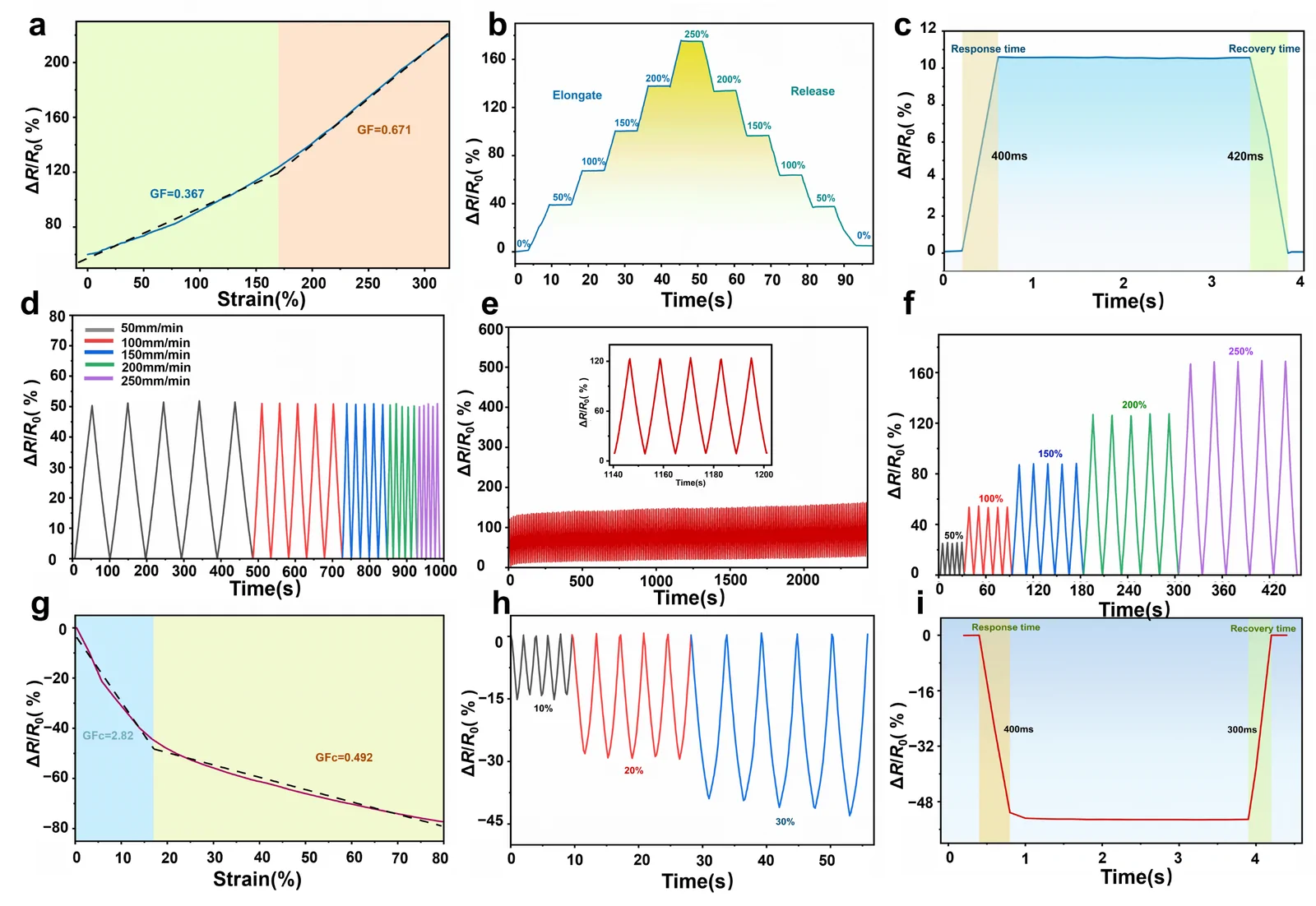
Strain-sensing performance of the optimized PCDGPL hydrogel. (**a**) Relative resistance change Δ*R*/*R*_0_ under 0–300% tensile strain. (**b**) Relative resistance response during stepwise tensile loading from 0% to 250% strain and subsequent release. (**c**) Response and recovery behavior under tensile loading and release. (**d**) Relative resistance responses at 100% strain under stretching rates of 50, 100, 150, 200, and 250 mm min^−1^. Gray, red, blue, green, and purple curves correspond to 50, 100, 150, 200, and 250 mm min^−1^, respectively. (**e**) Cyclic resistance response at a fixed tensile strain of 100%. (**f**) Repeated resistance responses under tensile strains of 50%, 100%, 150%, 200%, and 250%. (**g**) Relative resistance change under 0–80% compressive strain, with the dimensionless compressive gauge factor (GFc) determined from piecewise linear fitting. (**h**) Repeated resistance responses under compressive strains of 10%, 20%, and 30%. (**i**) Response and recovery behavior under compression and release. The sensing-response curves shown are representative results from three independent measurements with comparable response trends. In panels (**a**,**g**), the colored background regions indicate the strain ranges used for piecewise linear fitting, and the black dashed lines represent the corresponding linear fits. In panels (**c**,**i**), the shaded regions indicate the response and recovery intervals.

**Figure 5:**
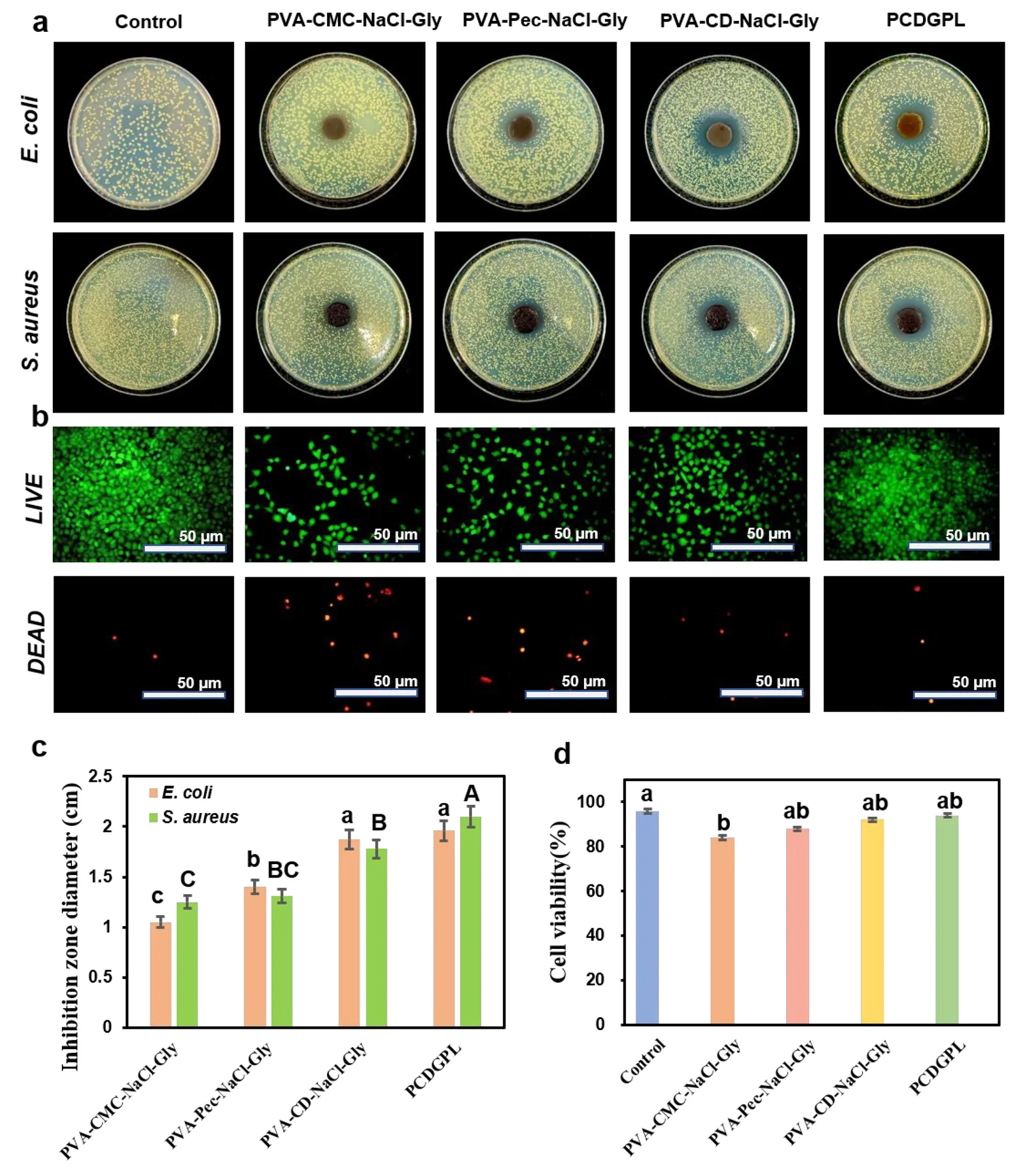
Antibacterial activity and cytocompatibility of different hydrogel films. (**a**) Inhibition zone assay against *E. coli* and *S. aureus*. (**b**) Representative live/dead fluorescence images of L929 fibroblasts treated with hydrogel extracts. (**c**) Total inhibition-zone diameters of the hydrogel formulations against *E. coli* and *S. aureus*. The reported diameter includes the hydrogel disc itself (diameter: 1.0 cm); therefore, a value close to 1.0 cm indicates negligible inhibition beyond the disc edge. The blank control without a hydrogel disc is shown only in panel (**a**) for qualitative comparison and was excluded from the quantitative analysis in panel (**c**). (**d**) Viability of L929 fibroblasts after treatment with hydrogel extracts for 24 h. Data in panels (**c**,**d**) are presented as mean ± SD (*n* = 3). In panel (**c**), different lowercase letters indicate statistically significant differences among formulations for *E. coli*, whereas different uppercase letters indicate statistically significant differences for *S. aureus*. In panel (**d**), different lowercase letters indicate statistically significant differences among groups. Different letters indicate statistical significance at *p* < 0.05 according to one-way ANOVA followed by Tukey’s post hoc test.

**Figure 6:**
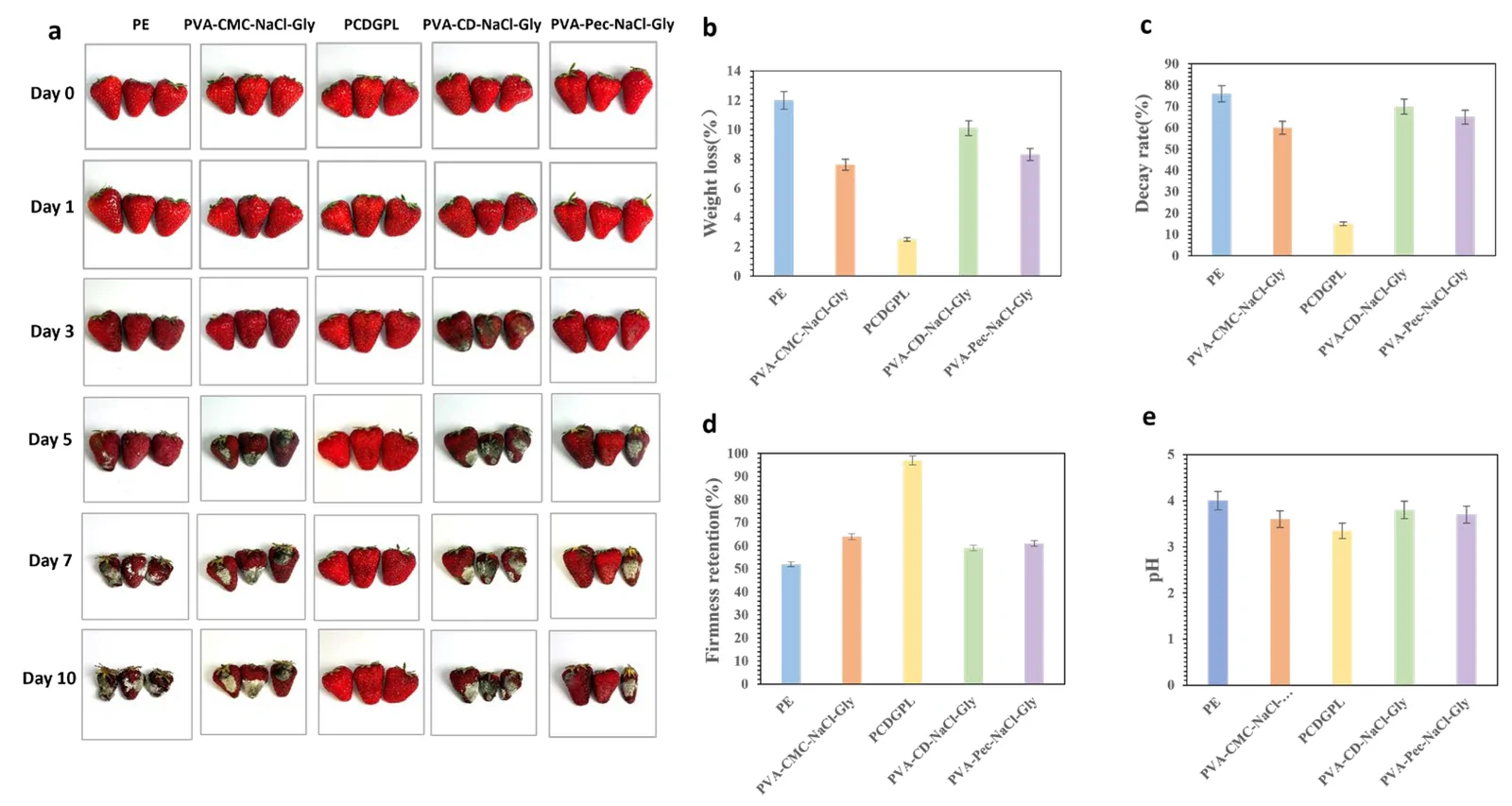
Strawberry preservation performance of different packaging films during storage. (**a**) Representative photographs of strawberries during storage. All samples were stored at approximately 25 °C under ambient relative humidity of approximately 43% RH; the relative humidity was not actively controlled. The rows correspond to storage days 0, 1, 3, 5, 7, and 10, while the columns correspond to the PE, PVA-CMC-NaCl-Gly, PCDGPL, PVA-CD-NaCl-Gly, and PVA-Pec-NaCl-Gly groups. The same row-and-column arrangement was intentionally maintained at all storage intervals to facilitate direct visual comparison among treatment groups and across storage intervals. Each image represents the corresponding treatment and storage interval, and no photograph was reused between subpanels. (**b**) Weight loss, (**c**) decay rate, (**d**) firmness retention, and (**e**) pH of strawberries on day 10. Each treatment group contained 30 strawberries divided into three independent replicates of 10 fruits each. The photographs show three representative strawberries from each treatment group at each storage interval. Data in panels (**b**–**e**) are presented as mean ± SD (*n* = 3). Different lowercase letters indicate statistically significant differences among treatment groups at *p* < 0.05 according to one-way ANOVA followed by Tukey’s post hoc test.

## Data Availability

The original contributions presented in this study are included in the article. Further inquiries can be directed to the corresponding author.

## Supplementary Materials

The following supporting information can be downloaded at: https://www.mdpi.com/article/10.3390/molecules31193377/s1, Figure S1: Effect of β-CD on the mechanical properties of the hydrogel films. (a) Representative tensile stress–strain curves and (b) representative compressive stress–strain curves of PCDGPL and its corresponding full-formulation-minus-β-CD control (PCDGPL-CD). All other components and preparation conditions were maintained unchanged; Figure S2: Moisture-related properties of the hydrogel films and optimized PCDGPL; Table S1: Composition and fabrication methods of representative multifunctional PVA hydrogels [25,26,27,28]; Table S2: Performance comparison of representative multifunctional PVA hydrogels [25,26,27,28].
