# Supplementary material for: Lysozyme-Functionalized PVA/CMC/β-Cyclodextrin/Pectin Hydrogel Films for Strawberry Preservation and Flexible Sensing
Source: Molecules. 2026 Sep 23;31(19):3377. doi: 10.3390/molecules31193377 (PMC13666277; doi:10.3390/molecules31193377)
Supplement: Supplementary file 1 [file molecules-31-03377-s001.zip › molecules-4504756-supplementary.pdf]

Supplementary Materials

Lysozyme-Functionalized PVA/CMC/β-Cyclodextrin/Pectin Hydrogel Films for Strawberry Preservation and Flexible Sensing

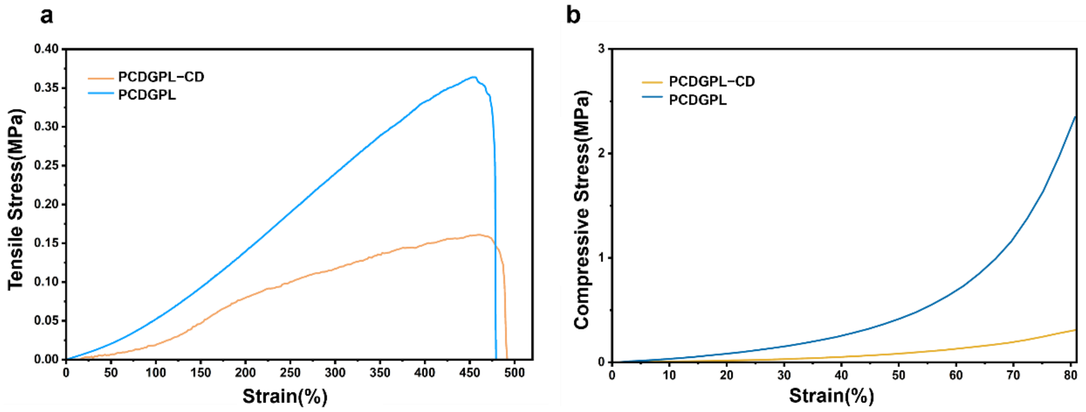

Figure S1. Effect of β-CD on the mechanical properties of the hydrogel films. (a) Representative tensile stress–strain curves and (b) representative compressive stress–strain curves of PCDGPL and its corresponding full-formulation-minus-β-CD control (PCDGPL−CD). All other components and preparation conditions were maintained unchanged.

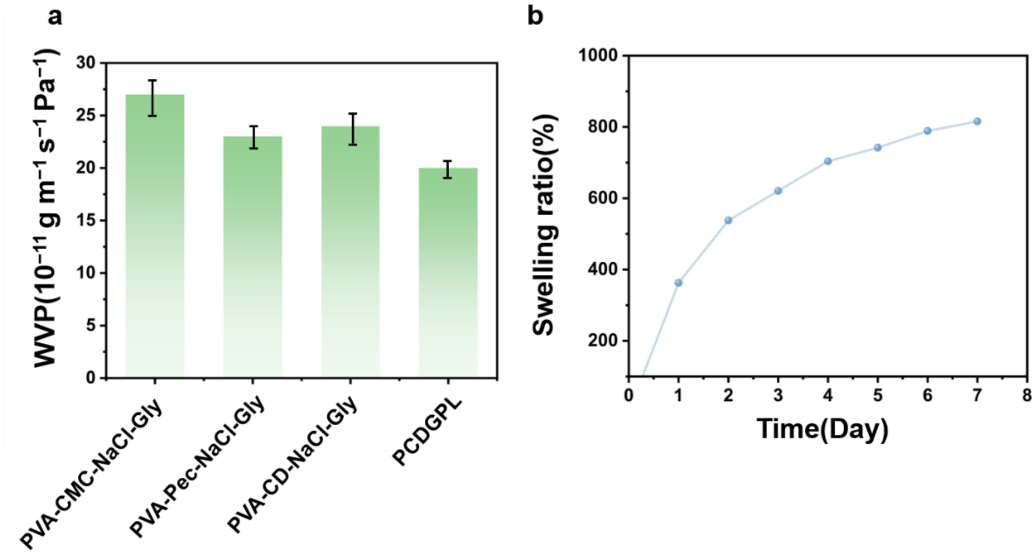

Figure S2. Moisture-related properties of the hydrogel films and optimized PCDGPL. (a) Water vapor permeability

of the different hydrogel films; (b) swelling ratio of PCDGPL during immersion in deionized water for 1–7 days. Data in panel (a) are presented as mean ± SD (n = 3).

Table S1. Composition and fabrication methods of representative multifunctional PVA hydrogels.

Study	Composition	Fabrication method
Gong et al. [25]	PVA/GA/Lyz	Solution blending and casting
Yu et al. [26]	SHNC/PVA/CS/Anth	Ultra-low-temperature cyclic freeze–thawing
Chen et al. [27]	PVA-p(AA-AM)-PA-Fe^{3+}	Photocrosslinking and PA–Fe^{3+} coordination
Fan et al. [28]	PVA/CS/β-CD derivative/NaCl	Freeze–thawing, Schiff-base DN and NaCl immersion
This work	PVA/CMC/Pec/β-CD/Gly/NaCl/Lyz	Five freeze–thaw cycles

Table S2. Performance comparison of representative multifunctional PVA hydrogels.

Study	Mechanical properties	Sensing	Antibacterial activity	Preservation
Gong et al. [25]	TS: 37.9 MPa	NR	*E. coli*, *S. aureus*	Blueberry: 20 d
Yu et al. [26]	TS: 26.84 MPa; EB: 345.26%	pH/NH_3 colorimetry; GF: NR	90.59%	Salmon: 6→14 d at 4 °C
Chen et al. [27]	TS: 0.12 MPa; EB: 1877%	GF: 2.14/1.67	Broad-spectrum	Strawberry: >10 d
Fan et al. [28]	TS: 0.50 MPa; EB: 314%	GF: 4.87	Reported	NR
This work	TS: ≈0.36 MPa; EB: ≈ 460–480%	GF: 0.367/0.671	*E. coli*, *S. aureus*	Strawberry: 10 d

TS, tensile strength; EB, elongation at break; GF, gauge factor; NR, not reported; GA, gallic acid; Lyz, lysozyme; CS, chitosan; Anth, anthocyanin.
